# Circulating oxysterols and prognosis among women with a breast cancer diagnosis: results from the MARIE patient cohort

**DOI:** 10.1186/s12916-023-03152-7

**Published:** 2023-11-14

**Authors:** Nina Sophia Decker, Theron Johnson, Johannes A. Vey, Charlotte Le Cornet, Sabine Behrens, Nadia Obi, Rudolf Kaaks, Jenny Chang-Claude, Renée Turzanski Fortner

**Affiliations:** 1 Division of Cancer Epidemiology, German Cancer Research Center (DKFZ), Im Neuenheimer Feld 280, 69120 Heidelberg, Germany; 2 Medical Faculty Heidelberg, Heidelberg University, Heidelberg, Germany; 3 Institute of Medical Biometry, Heidelberg University, Heidelberg, Germany; 4 Institute of Medical Biometry and Epidemiology, University Medical Center Hamburg-Eppendorf, Martinistrasse 52, 20246 Hamburg, Germany; 5 University Cancer Center Hamburg, Medical Center Hamburg-Eppendorf, Martinistrasse 52, 20246 Hamburg, Germany; 6 Department of Research, Cancer Registry of Norway, Ullernchausseen 64, 0379 Oslo, Norway

**Keywords:** Oxysterols, Cholesterol metabolism, Breast cancer, Survival, Cardiovascular death, Cancer death, Cohort study, Molecular epidemiology

## Abstract

**Background:**

Breast cancer is the most commonly diagnosed cancer in women worldwide, and underlying mechanistic pathways associated with breast cancer-specific and non-breast cancer-related deaths are of importance. Emerging evidence suggests a role of oxysterols, derivates of cholesterol, in multiple chronic diseases including breast cancer and coronary artery diseases. However, associations between oxysterols and survival have been minimally studied in women diagnosed with breast cancer. In this large breast cancer patient cohort, we evaluated associations between a panel of circulating oxysterols and mortality and recurrence outcomes.

**Methods:**

Concentrations of 13 circulating oxysterols representing different pathways of cholesterol metabolism were quantified using liquid-chromatography mass-spectrometry. Associations between baseline levels of oxysterols and cause-specific mortality outcomes and recurrence following a breast cancer diagnosis were assessed in 2282 women from the MARIE study over a median follow-up time of 11 years. We calculated hazard ratios (HR) and 95% confidence intervals (CI) using multivariable Cox proportional hazard models and competing risks models.

**Results:**

We observed no associations for circulating oxysterols and breast cancer-specific outcomes. Higher levels of six oxysterols were associated with an increased risk of cardiovascular disease death, including 24S-hydroxycholesterol (alternative bile acid pathway, HR_log2_ = 1.73 (1.02, 2.93)), lanosterol (cholesterol biosynthesis pathway, HR_log2_ = 1.95 (1.34, 2.83)), 7-ketocholesterol (HR_log2_ = 1.26 (1.03, 1.55)), 5α,6α-epoxycholesterol (HR_log2_ = 1.34 (1.02–1.77)), and 5a,6β-dihydroxycholestanol (HR_log2_ = 1.34 (1.03, 1.76)). After adjusting for multiple comparisons, none of the associations were statistically significant.

**Conclusion:**

We provide first evidence on a range of circulating oxysterols and mortality following a breast cancer diagnosis, contributing to a better understanding of associations between different pathways of cholesterol metabolism and prognosis in women with a breast cancer diagnosis. The findings of this study suggest circulating oxysterols may be associated with cardiovascular mortality among women diagnosed with breast cancer. Further studies are needed to evaluate these oxysterols as potential markers of risk for cardiovascular mortality among women with a breast cancer diagnosis as well as their clinical potential.

**Supplementary Information:**

The online version contains supplementary material available at 10.1186/s12916-023-03152-7.

## Background

Breast cancer is the most commonly diagnosed cancer in women worldwide with a total of 2.3 million new cases in 2020 [1]. Due to improvements in breast cancer diagnosis and treatment, non-breast cancer-related deaths are of increasing importance in breast cancer survivors [2]. A recent population-based study in the United States reported that deaths from cardiac causes (10%) and other cancer deaths (6.7%) are the second and third most common causes of death among women diagnosed with breast cancer [3]. With survival beyond 10 years after initial breast cancer diagnosis, the majority of these breast cancer survivors died of causes other than breast cancer [3]. Furthermore, studies showed that women with a breast cancer diagnosis are at greater risk for cardiovascular disease mortality as compared to women without breast cancer [4, 5]. However, understanding of pathways associated with causes of death other than cancer in breast cancer survivors is limited [4].

Emerging evidence suggests that oxysterols may play a role in multiple chronic diseases including breast cancer [6, 7], coronary artery diseases [7, 8], and neurodegenerative disease [9]. Oxysterols are produced as intermediates in metabolic processes including cholesterol removal from brain [10–12] or bile acid biosynthesis [7]; they can also act as precursors of cholesterol [13, 14]. Oxysterols have been shown to exert functional and regulatory roles in lipid metabolism and signaling [15, 16]. We [17, 18] and others [19] have previously investigated circulating 27-hydroxycholesterol (HC), and 25-HC with respect to breast cancer risk and survival due to their reported activity as estrogen receptor modulators. Associations between other analytes of the cholesterol metabolism and breast cancer in humans have been minimally investigated.

In this study, we aimed to explore the associations between a panel of oxysterols, representing different upstream and downstream cholesterol metabolism pathways, and survival and breast cancer recurrence in a large cohort of women recruited following a breast cancer diagnosis. We evaluated overall survival and breast cancer-specific survival and, due to the reported effects of oxysterols in other chronic diseases and the relatively high reported frequency of non-breast cancer causes of death among breast cancer patients, the further endpoints of (a) other cancer death, (b) cardiovascular death, and (c) death due to other causes.

## Materials and methods

### Study sample and data collection

This study was conducted in the Mammary Carcinoma Risk Factor Investigation (MARIE) breast cancer patient cohort, which has been described in detail previously [20]. In brief, 3813 women with a breast cancer diagnosis were initially enrolled between August 2002 and September 2005 in two regions (Rhine-Neckar-Karlsruhe and Hamburg) in Germany. Participants were aged 50–74 years at baseline and had a histologically confirmed primarily invasive or *in situ* carcinoma diagnosis. At recruitment, participants completed an in-person interview and provided data on lifestyle, health, and anthropometric characteristics and were requested to provide a blood sample. Follow-up interviews were conducted in 2009 (first follow-up) and in 2015 (second follow-up). In the present study, participants with *in situ* breast cancers, metastasis at diagnosis/stage IV breast cancer, previous tumors other than breast cancer, unknown stage, or missing hormone receptor status were excluded. A total of 2282 participants with available blood sample were included in this study [18]. Main study characteristics such as age and BMI were largely similar between cases of the overall cohort [20] and the study population investigated here; stage at diagnosis differed by design excluding women with metastasis at diagnosis or *in situ* breast cancer who were part of the original study sample. Eight participants with blood collection before breast cancer diagnosis due to original recruitment as “control” were then re-classification as “case” (median time between blood collection and diagnosis: 5.3 months (range 1.6 months to 14.7 months)); exclusion of these participants (0.4% of the study sample) did not impact the observed associations and therefore these cases were retained in the study. The median time between diagnosis and blood collection for the full study sample was 3.7 months (range − 14.7 months to 57.6 months). Participants of the MARIE study were asked about their last natural menstrual period at baseline and were defined as postmenopausal if the reference date was at least one year after their last natural menstrual period or if they had bilateral oophorectomy or had cessation of menstrual period because of radiation or chemotherapy for a disease other than breast cancer. Participants older than 55 years with unclear menopausal status because of hysterectomy or menopausal hormone therapy were also defined as postmenopausal in the MARIE study [20]. The use of exogenous selective estrogen receptor modulators (SERMs) such as tamoxifen and aromatase inhibitor (AI) was assessed via questionnaires at both follow-up times for the preceding time interval. If self-reported information on endocrine therapy was not available, information derived from medical records was used. A description of the sample selection of this study as well as detailed information on endocrine therapy use has been reported previously [18].

### Ascertainment of clinical outcomes

Follow-up information was obtained from participants by telephone interview at each follow-up, and recurrence information was confirmed through medical records or contact with treating physicians. Vital status was obtained from population registries of the study regions up to the end of the second follow-up period in 2015, and copies of the death certificates were obtained from local health offices. Causes of death were coded according to the 10th revision of the International Classification of Diseases (ICD-10-WHO).

The outcomes for this study included all-cause mortality, cause-specific mortality (breast cancer death, other cancer death, cardiovascular disease death, other cause of death), and risk of breast cancer recurrence. “All-cause mortality” was attributed to death by any cause, “breast cancer (BC)-specific mortality” was attributed to breast cancer deaths (ICD-10-C50), and “recurrence” was defined using the definition for recurrence-free interval as described in the Standardized Definitions for Efficacy End Points (STEEP) criteria [21] and includes local/regional invasive breast cancer recurrence, metastasis, contralateral disease of the breast, and deaths due to breast cancer. This study includes 438 all-cause deaths, 237 BC-specific deaths, and 376 recurrences. The outcome “other cancer death” was assigned to all cancer deaths (ICD-10-C) other than breast cancer including cancer of digestive organs (*n* = 30), respiratory organs (*n* = 23), genital organs (*n* = 9), and other cancers (*n* = 26, *n* ≤ 5 for any other individual cancer); “cardiovascular deaths” was assigned to all deaths of the cardiovascular system (ICD-10-I) including deaths due to acute myocardial infarction (*n*=13), heart failure (*n*=10), chronic ischemic heart disease (*n* = 8), pulmonary embolism (*n* = 6), and other (*n* = 24; *n*≤6 for any other individual cardiovascular death); “other cause of death” includes all of the deaths not attributed to any of the other outcomes including death due to the respiratory system (ICD-10-J, *n* = 16), digestive system (ICD-10-K, *n* = 9), infectious and parasitic diseases (ICD-10-A/B, *n* = 8), nervous system (ICD-10-G, *n* = 6), and other (*n* = 13; *n* ≤ 6 for any other individual cause of death).

### Laboratory

The following oxysterols were considered for inclusion in this study: 22R-hydroxycholesterol (22R-HC), 24S-hydroxycholesterol (24S-HC), 5α,6α-epoxycholesterol (5a,6a-EC), 5β,6β-epoxycholesterol (5b,6b-EC), 7α-hydroxycholesterol (7a-HC), 7β-hydroxycholesterol (7b-HC), 7-dehydrocholesterol (7-DC), 7-ketocholesterol (7-KC), desmosterol (desmos), lanosterol (lan), 24,25-dihydrolanosterol (24-DHLan), 24,25-epoxycholesterol (24,25-EC), 5α,6β-dihydroxycholestanol (THC). Oxysterols 27-hydroxycholesterol (27-HC; systematic name (25R),26-hydroxycholesterol) and 25-hydroxycholesterol (25-HC), and estradiol have been investigated in a previous study [18] and were evaluated as potential covariates in the current study. Oxysterol levels were measured by biocrates life sciences ag (Innsbruck, Austria) using UHPLC-MS/MS with multiple reaction monitoring (MRM) in positive mode using a mass spectrometer with electrospray ionization (ESI). Inter-assay coefficients of variation (CV) were determined by including 16 blinded replicate quality controls. Analyte concentrations and coefficients of variation (CV) are displayed in the additional file (Additional file 1, Table S1). In brief, mean intra-assay CVs were below 20% (Additional file 1, Table S1), and mean inter-assay CVs were below 25%, with the exception of 5a6a-EC (31.8%), 24-DHLan (33.7%), 7-KC (42.4%), 7b-HC (74.9%). 7b-HC was excluded from the survival analyses due to the high CV (74.9%). Of note, CVs were highest for oxysterols with low concentrations (median concentrations of oxysterols with CV > 30% are between 4.8 and 34.8 nM, whereas median concentrations of oxysterols with CV ≤ 30% are between 47.2 nM and 2705.8 nM), and median concentrations of quality controls with CV > 30% were lower in the quality control samples than median concentrations measured in the study samples (e.g., median, 7b-HC, QC samples: 5.8 nM, participant samples: 202.3 nM; 7-KC, QC samples: 34.8 nM, participant samples: 184.9 nM). Estradiol concentrations were measured using an ELISA in the Division of Cancer Epidemiology at the German Cancer Research Center (DKFZ) (inter-batch CV of 16.2%).

In the following, the term “oxysterol” is attributed to cholesterol metabolites (24S-HC, 7a-HC, 7b-HC, 7-KC, THC, 5a6a-EC, 5b6b-EC, 22R-HC, 27-HC, 25-HC) as well as precursors of cholesterol (lanosterol, 24-DHLan, desmosterol, 7-DC, 24,25-EC).

### Statistics

We applied a log_2_-transformation to all biomarker concentrations to obtain approximately normal biomarker distributions and to estimate the effect of a doubling in biomarker concentrations.

Values below the limit of detection (LOD) were detected for the following biomarkers: 24S-HC, *n* = 7; 7b-HC, *n* = 14; desmosterol, *n* = 11; 7-DC, *n* = 150; THC, *n* = 492; 25-HC, *n* = 89; estradiol, *n*=12 (Additional file 1, Table S1). We imputed these values with the midpoint between 0 and the lowest detectable value stratified by the study region. 22R-HC and 24,25-EC were excluded from all analyses due to the high number of values below LOD (> 88%). Values exceeding the calibration range were detected for the following biomarkers: 24S-HC, *n* = 2; 5a6a-EC, *n* = 1; 5b6b-EC, *n* = 4; 7a-HC, *n* = 11; 7b-HC, *n* = 10; desmosterol, *n* = 1; 7-DC, *n* = 61 (Additional file 1, Table S1); these values were excluded from all analyses. After imputing values below LOD and excluding values exceeding the calibration range, we evaluated outlying values using the Generalized ESD Many-Outlier Procedure [22]. Overall, ≤ 30 outliers were detected for any biomarker, and analyses were conducted with and without inclusion of the outliers.

We calculated Spearman partial correlations for circulating oxysterols adjusted for age at diagnosis and study region. To account for the left-truncated survival data, delayed-entry Cox proportional hazards regression was used to estimate hazard ratios (HR) and 95% confidence intervals (CI) for risk of all-cause mortality. HRs were calculated using continuous log_2_-transformed oxysterol values. We assessed associations between each individual oxysterol and cause-specific mortality (BC-specific death, other cancer death, cardiovascular death, and death due to other causes) and risk of recurrence (recurrence vs. non-breast cancer cause of death) using the competing risks approach described by Lunn and McNeil [23].

Non-parametric restricted cubic splines were used to examine possible non-linearity, comparing models with linear and cubic terms to models with only the linear term [24]. We did not observe significant deviation from linearity. The start date for follow-up time (time-to-event) was the date of diagnosis and the start date for participants at risk (time-at-risk) was the date of blood collection. End of follow-up time was the date of the defined outcome, the date of last contact, or the end of follow-up 2 (June 2015), whichever came first. Associations were evaluated for all participants and by hormone receptor status (ER/progesterone receptor (PR)-positive and ER/PR-negative). In the ER/PR-negative subset, only the outcomes all-cause death, BC-specific death, and recurrences were evaluated due to limited sample size (*n* = 320).

Covariates for all of the models were selected *a priori* and included: age at diagnosis, body mass index (BMI), smoking status (never, former, current smoker), alcohol consumption (g/day), Charlson Comorbidity Index (CCI), and tumor size (≤ 2 cm, 2–5 cm, > 5 cm), nodal status (0, 1–3, >3), and tumor grade (low, moderate, high). All models were stratified by ER/PR-status (ER+/PR+, ER+/PR− or ER−/PR+, ER−/PR−), and study region (Hamburg, Rhine-Neckar-Karlsruhe). The biomarkers 27-HC, 25-HC, and estradiol, as well as physical activity, parity, and educational status were considered as further potential covariates; however, adding these covariates to the model minimally changed the results (< 10 %) and they were excluded from the final models. Of the evaluated oxysterols, 7-DC was strongly associated with endocrine therapy (endocrine therapy vs. no endocrine therapy, 16% difference) and THC was strongly associated with endocrine therapy and chemotherapy (endocrine therapy vs. no endocrine therapy, -18% difference; chemotherapy vs. no chemotherapy, 29% difference). In further analyses, we adjusted associations with these oxysterols additionally for chemotherapy or endocrine therapy. Controlling for smoking status using a continuous variable (duration of smoking in years or average pack years of smoking) resulted in similar results as use of categorical variables smoking status (never, former, current), which we used due to completeness of data. Study participants with missing information for any of the covariates were excluded (*n* participants = 17). The proportional hazards assumption was tested using Schoenfeld residuals; we did not observe violation of the proportional hazards assumption.

In an exploratory analysis, we applied a regularized Cox proportional hazard regression model using the elastic net penalty, a combination between LASSO and Ridge regression [25], controlling for the covariates listed above with the aim to select variables strongest associated with the outcomes. Hyper-parameter tuning was performed to identify the best α (elastic net mixing parameter) and λ (shrinkage parameter) applying 5-fold cross-validation and optimizing the C-index. In a sensitivity analysis, we conducted analyses including biomarkers with low CV (< 30%) only. Associations between the selected oxysterols and the outcomes were evaluated in competing risks models. In further sensitivity analyses, we conducted stratified 5-year survival analyses. Finally, we evaluated the effect of adjusting for multiple comparisons (60 comparisons in our main analysis for 6 outcomes and 10 oxysterols).

All statistical tests were two-tailed and considered significant at *p* < 0.05. Statistical analyses were conducted using SAS 9.4 (SAS Institute Inc., Cary, NC, USA) and R (version 4.2.1).

## Results

### Baseline characteristics

Among the overall cohort (*n* = 2282), at follow-up 2015, 436 deaths were observed with nearly half of the deaths attributed to breast cancer (*n* = 237; 54.1%), followed by other cancer death (*n* = 88; 20.1%), cardiovascular death (*n* = 61; 13.9%), and death due to other causes (*n* = 52; 11.9%) (Table 1).

**Table 1:** Study characteristics by cause of death, median (range), or %: MARIE patient cohort

		**Outcome status at end of follow-up**				
**Study characteristics**	**Overall cohort**	**Alive**	**BC-specific death**	**Other cancer death**	**Cardiovascular death**	**Other cause of death**
*N*, total	2282	1844	237	88	61	52
Median FU time (years)	11.2 (0.3–14.5)	11.6 (9.7, 14.5)	5.9 (0.3, 13.4)	8.1 (1.1, 13.1)	7.5 (0.4, 12.3)	7.6 (0.3, 13.2)
Age at diagnosis (years)	63.0 (50.0–75.0)	62.6 (50.0, 75.0)	63.5 (50.2, 74.7)	64.9 (50.6, 74.8)	67.8 (51.8, 74.6)	65.1 (52.4, 74.9)
BMI (continuous) in kg/m^2^	25.3 (15.5–49.6)	25.2 (15.5, 49.6)	26.3 (17.4, 43.1)	25.6 (16.3, 40.2)	27.0 (17.2, 47.8)	24.8 (17.3, 39.5)
Smoking						
Never	1239 (54.3%)	1006 (54.6%)	135 (57.0%)	35 (39.8%)	35 (57.4%)	28 (53.9%)
Former	615 (27.0%)	508 (27.6%)	55 (23.2%)	28 (31.8%)	16 (26.2%)	8 (15.4%)
Current	428 (18.8%)	330 (17.9%)	47 (19.8%)	25 (28.4%)	10 (16.4%)	16 (30.8%)
Alcohol consumption in g/day						
0	540 (23.7%)	407 (22.1%)	69 (29.1%)	24 (27.6%)	22 (36.1%)	18 (34.6%)
1–5	845 (37.1%)	683 (37.1%)	91 (38.4%)	33 (37.9%)	24 (39.3%)	14 (26.9%)
5–19	560 (24.6%)	466 (25.3%)	51 (21.5%)	21 (24.1%)	9 (14.8%)	13 (25.0%)
≥ 19	335 (14.7%)	287 (15.6%)	26 (11.0%)	9 (10.3%)	6 (9.8%)	7 (13.5%)
Charlson Comorbidity Index						
0	1487 (65.2%)	1246 (67.57%)	145 (61.2%)	40 (45.5%)	29 (47.5%)	27 (51.9%)
1	513 (22.5%)	399 (21.64%)	56 (23.6%)	28 (31.8%)	15 (24.6%)	15 (28.9%)
2+	282 (12.4%)	199 (10.79%)	36 (15.2%)	20 (22.7%)	17 (27.9%)	10 (19.2%)
ER/PR status						
ER+/PR+	1583 (69.4%)	1307 (70.88%)	128 (54.0%)	67 (76.1%)	44 (72.1%)	37 (71.2%)
ER+/PR− or ER−/PR+	376 (16.5%)	303 (16.43%)	51 (21.5%)	6 (6.8%)	7 (11.5%)	9 (17.3%)
ER−/PR−	323 (14.2%)	234 (12.69%)	58 (24.5%)	15 (17.1%)	10 (16.4%)	6 (11.5%)
HER2/neu status						
HER2+	444 (19.5%)	340 (18.4%)	62 (26.2%)	16 (18.2%)	17 (27.9%)	9 (17.3%)
HER2−	1717 (75.2%)	1410 (76.5%)	164 (69.2%)	64 (72.7%)	41 (67.2%)	38 (73.1%)
Unknown	121 (5.3%)	94 (5.1%)	11 (4.6%)	8 (9.1%)	3 (4.9%)	5 (9.6%)
Breast cancer stage at diagnosis						
I	1137 (49.8%)	987 (53.52%)	56 (23.6%)	42 (47.7%)	29 (47.5%)	23 (44.2%)
IIa	718 (31.5%)	575 (31.18%)	70 (29.5%)	33 (37.5%)	23 (37.7%)	17 (32.7%)
IIb	273 (12.0%)	182 (9.87%)	68 (28.7%)	10 (11.4%)	6 (9.8%)	7 (13.5%)
IIIa	154 (6.8%)	100 (5.42%)	43 (18.1%)	3 (3.4%)	3 (4.9%)	5 (9.6%)

*Abbreviations*: *FU* Follow-up time, *BMI* Body mass index, *ER* Estrogen receptor, *PR* Progesterone receptor, *HER2* Human epidermal growth factor 2

Missings values: BMI *n* = 3; alcohol consumption *n* = 2

Median follow-up time was longest among participants alive at the end of follow-up (11.6 years), and shortest among participants who died due to breast cancer (5.9 years). The median age at breast cancer diagnosis of the cohort was 63 years (range: 50–75 years). Participants with cardiovascular death had the highest median age at breast cancer diagnosis (67.8 years), while women with BC-specific death had the lowest (63.5 years). Participants with other cancer-related deaths and other cause of death had the highest proportions of smokers (28.4% and 30.8%, respectively), as compared to 18.8% in the full cohort. A lower proportion of participants reported high alcohol consumption (≥ 19 g/day) in other cancer death (10.3%) and cardiovascular death (9.8%) groups, compared to the overall cohort (14.7%). Higher Charlson Comorbidity Index scores (i.e., 2+) were observed in the cardiovascular death group (27.9%) as compared to the full cohort (12.4%). BMI was similar between groups, with a median of 25.3 km/m^2^ (range: 15.5–49.6 km/m^2^) in the overall study population.

A majority of participants had ER- and PR-positive tumors (69.4%), 16.5% had discordant ER and PR tumors, and 14.2% had hormone-receptor-negative tumors. Participants who died due to breast cancer had a higher proportion of hormone-receptor-negative breast cancer (24.5%). The majority of the study population was diagnosed with stage I (49.8%) or stage IIa (31.5%) breast cancer; participants who died due to breast cancer had a higher breast cancer stage at diagnosis (stage I: 23.6%; stage IIa: 29.5%). The majority of participants were postmenopausal at diagnosis (91.0%), while 9.0% were peri-menopausal or of unknown menopausal status due to hysterectomy or hormone replacement therapy.

### Correlations

**Fig. 1:**
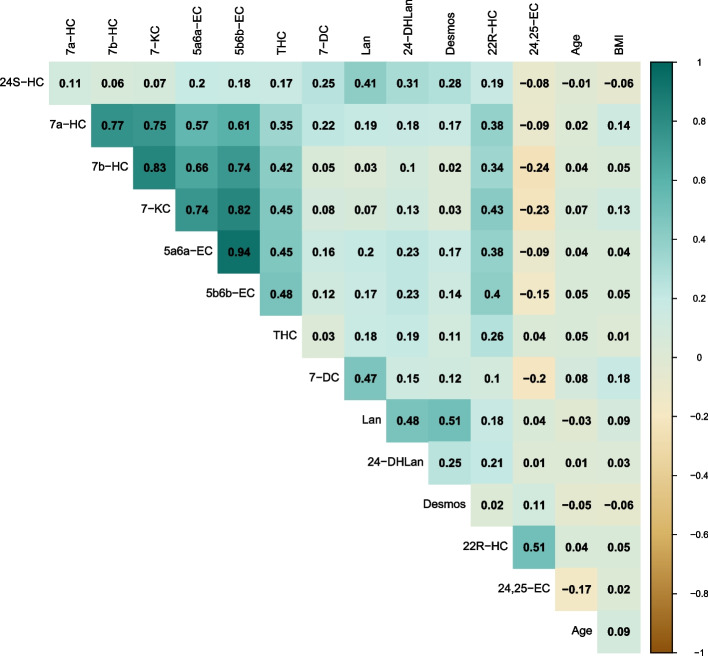
Correlation matrix of circulating oxysterols: MARIE patient cohort. Spearman partial correlation coefficients, adjusted for age and center; using imputed, log_2_-transformed values. *n* = 2269, except 7-DC (*n* = 2209), 24-DHLan (*n* = 1019), 22R-HC (*n* = 263), 24,25-EC (*n* = 86). Abbreviations: 24S-HC, 24S-hydroxycholesterol; 7a-HC, 7α-hydroxycholesterol; 7b-HC, 7β-hydroxycholesterol; 7-KC, 7-ketocholesterol; 5a6a-EC, 5α,6α-epoxycholesterol; 5b6b-EC, 5β,6β-epoxycholesterol; THC, 5α,6β-dihydroxycholestanol; 7-DC, 7-dehydrocholesterol; Lan, lanosterol; 24-DHLan, 24,25-dihydrolanosterol; Desmos, desmosterol; 22R-HC, 22R-hydroxycholesterol; 24,25-EC, 24,25-epoxycholesterol; BMI, body mass index

We observed strong correlations between 7a-HC, 7b-HC, and 7-KC (0.75 ≥ *r* ≥ 0.83); 5a6a-EC and 5b6b-EC were strongly correlated with each other (*r* = 0.94), and moderately to strongly correlated with the group of 7a-HC, 7b-HC, and 7-KC (0.57 ≥ *r* ≥ 0.82) (Fig. 1). THC was moderately correlated with these oxysterols (0.35 ≥ *r* ≥ 0.48). Lanosterol was moderately correlated with 24S-HC (*r* = 0.41), 7-DC (*r* = 0.47), 24-DHLan (*r* = 0.48), and desmosterol (*r* = 0.51). 22R-HC was moderately correlated with 7-KC (*r* = 0.43) and with 24,25-EC (*r* = 0.51). Correlation coefficients between the other oxysterols were weaker (*r* ≤│0.40│). The oxysterols were weakly associated with age and BMI (*r* <│0.18│) (Fig. 1), and estradiol (*r* ≤│0.29│) (Additional file 1, Table S2). 24S-HC and lanosterol were modestly correlated with 27-HC (*r* = 0.51 and *r* = 0.42, respectively), and 7a-HC, 7b-HC, 7-KC, 5a6a-EC, and 5b6b-EC were modestly correlated with 25-HC (0.44 ≥ *r* ≥ 0.50), while correlations between 27-HC and 25-HC and the other oxysterols were weaker (*r* ≤│0.39│) (Additional file 1, Table S2).

### Associations between oxysterols and recurrence and survival

We observed no associations between circulating oxysterols and all-cause death, BC-specific death, and risk of recurrences (Table 2). A doubling of 24S-HC (HR_log2_ = 1.73 (1.02, 2.93)), 7-KC (HR_log2_ = 1.26 (1.03, 1.55)), 5a6a-EC (HR_log2_ = 1.34 (1.02, 1.77)), 5b6b-EC (HR_log2_ = 1.47 (1.10, 1.98)), THC (HR_log2_ = 1.34 (1.03, 1.76)), and lanosterol (HR_log2_ = 1.95 (1.34,2.83)) was associated with an increased risk of cardiovascular death. HRs and 95% CI of 7-KC, 5a6a-EC, 5b6b-EC, THC, and lanosterol were similar to the overall results in the hormone receptor-positive subgroup, while the magnitude of effect for 24S-HC was attenuated in cases with hormone receptor-positive disease. No associations were observed for oxysterols and all-cause death, BC-specific death, and risk of recurrence in hormone-receptor-negative patients (Additional file 1, Table S3), except for THC and risk of recurrence (HR_log2_ = 1.38 (1.05, 1.81)). Sample size precluded an evaluation of the other outcomes.

**Table 2:** Oxysterols and survival and recurrence following a breast cancer diagnosis: MARIE patient cohort

			Competing risks: causes of death				Competing risks: recurrences
		**All-cause death**	**BC-specific death**	**Other cancer death**	**Cardiovascular death**	**Other death**	**Recurrences**
		HR (95% CI)*	**HR (95% CI)**	**HR (95% CI)**	**HR (95% CI)**	**HR (95% CI)**	**HR (95% CI)**
*n* events (all/ ER/PR+)	*N* total	*434/346*	*236/178*	*87/73*	*60/50*	*51/45*	*373/288*
***Cholesterol metabolites***							
**24S-HC**	*2263*	1.11 (0.91, 1.36)	1.23 (0.94, 1.61)	0.76 (0.53, 1.10)	1.73 (1.02, 2.93)	0.79 (0.49, 1.29)	1.14 (0.92, 1.40)
ER/PR-positive	*1943*	1.11 (0.89, 1.39)	1.31 (0.98, 1.75)	0.76 (0.51, 1.13)	1.51 (0.85, 2.70)	0.77 (0.45, 1.33)	1.06 (0.85, 1.33)
**7a-HC**	*2254*	0.98 (0.87, 1.10)	0.95 (0.82, 1.12)	0.87 (0.67, 1.14)	1.22 (0.89, 1.68)	1.03 (0.73, 1.47)	0.90 (0.79, 1.03)
ER/PR-positive	*1935*	1.06 (0.93, 1.21)	1.03 (0.86, 1.23)	0.95 (0.71, 1.26)	1.30 (0.92, 1.83)	1.10 (0.77, 1.59)	0.89 (0.77, 1.03)
**7-KC**	*2265*	1.02 (0.95, 1.11)	0.97 (0.88, 1.08)	0.98 (0.82, 1.17)	1.26 (1.03, 1.55)	1.06 (0.84, 1.33)	0.95 (0.87, 1.03)
ER/PR-positive	*1945*	1.06 (0.98, 1.16)	1.00 (0.89, 1.13)	1.02 (0.84, 1.24)	1.31 (1.05, 1.63)	1.08 (0.85, 1.37)	0.93 (0.84, 1.02)
**5a6a-EC**	*2264*	1.04 (0.93, 1.16)	0.98 (0.83, 1.14)	1.02 (0.79, 1.33)	1.34 (1.02, 1.77)	0.97 (0.68, 1.39)	0.96 (0.85, 1.09)
ER/PR-positive	*1944*	1.04 (0.92, 1.18)	0.96 (0.81, 1.15)	1.00 (0.75, 1.34)	1.41 (1.06, 1.88)	0.99 (0.68, 1.44)	0.90 (0.78, 1.04)
**5b6b-EC**	*2261*	1.07 (0.95, 1.19)	0.97 (0.83, 1.14)	1.03 (0.80, 1.34)	1.47 (1.10, 1.98)	1.07 (0.76, 1.51)	0.96 (0.85, 1.09)
ER/PR-positive	*1941*	1.10 (0.97, 1.25)	0.97 (0.81, 1.16)	1.07 (0.80, 1.42)	1.57 (1.15, 2.16)	1.16 (0.81, 1.67)	0.91 (0.79, 1.06)
**THC**	*2265*	1.04 (0.98, 1.09)	1.04 (0.90, 1.22)	1.08 (0.84, 1.39)	1.34 (1.03, 1.76)	1.08 (0.78, 1.50)	1.00 (0.89, 1.13)
ER/PR-positive	*1945*	1.03 (0.98, 1.10)	1.00 (0.85, 1.19)	1.16 (0.89, 1.50)	1.34 (1.00, 1.79)	1.03 (0.72, 1.46)	0.94 (0.81, 1.08)
***Cholesterol precursors***							
**7-DC**	*2205*	0.99 (0.93, 1.05)	0.97 (0.92, 1.03)	0.97 (0.89, 1.06)	1.03 (0.90, 1.18)	0.95 (0.85, 1.06)	0.96 (0.92, 1.00)
ER/PR-positive	*1892*	0.99 (0.92, 1.06)	0.95 (0.89, 1.01)	0.98 (0.88, 1.09)	1.08 (0.91, 1.28)	0.97 (0.85, 1.11)	0.95 (0.90, 1.00)
**Lan**	*2265*	0.98 (0.85, 1.12)	1.00 (0.83, 1.21)	0.72 (0.53, 0.97)	1.95 (1.34, 2.83)	0.70 (0.48, 1.03)	1.01 (0.87, 1.18)
ER/PR-positive	*1945*	0.97 (0.83, 1.13)	1.04 (0.83, 1.30)	0.70 (0.50, 0.98)	2.13 (1.40, 3.25)	0.65 (0.43, 0.98)	1.05 (0.88, 1.26)
**24-DHLan**	*1023*	0.95 (0.80, 1.14)	0.92 (0.72, 1.17)	0.95 (0.64, 1.42)	1.06 (0.65, 1.73)	0.84 (0.51, 1.38)	0.97 (0.80, 1.18)
ER/PR-positive	*862*	0.99 (0.81, 1.21)	0.94 (0.71, 1.24)	1.09 (0.70, 1.70)	1.18 (0.68, 2.03)	0.79 (0.48, 1.33)	1.04 (0.83, 1.31)
**Desmos**	*2264*	1.00 (0.88, 1.12)	1.06 (0.92, 1.23)	1.02 (0.81, 1.27)	0.95 (0.76, 1.19)	0.86 (0.73, 1.02)	1.12 (0.99, 1.26)
ER/PR-positive	*1944*	0.94 (0.82, 1.07)	1.02 (0.87, 1.21)	0.98 (0.79, 1.21)	1.02 (0.76, 1.36)	0.83 (0.71, 0.98)	1.08 (0.95, 1.22)

^*^Hazard ratios (HR) and 95% confidence intervals (95% CI) for all-cause mortality from Cox proportional hazard models. All other HR and 95% CI from competing risks models. All models are adjusted for age at diagnosis, BMI, tumor size, nodal status, histological grading, smoking status (never, former, current), alcohol consumption, Charlson Comorbidity Index (CCI), and stratified by study region and ER/PR status. Oxysterol values are log_2_-transformed. *P*-values for significant associations between 0.01 and 0.05. After Bonferroni-Correction (*p*<0.0025), none of the results is statistically significant. Abbreviations: *24S-HC*, 24S-hydroxycholesterol; *22R-HC*, 22R-hydroxycholesterol; *5a6a-EC*, 5α,6α-epoxycholesterol; *5b6b-EC*, 5β,6β-epoxycholesterol; *7-KC*, 7-ketocholesterol; *7a-HC*, 7α-hydroxycholesterol; Lan, lanosterol; *24-DHLan*, 24,25-dihydrolanosterol; *7-DC*, 7-dehydrocholesterol; *Desmos*, desmosterol; *THC*, 5α,6β-dihydroxycholestanol

Inverse associations were observed between lanosterol and other cancer deaths (HR_log2_ = 0.72 (0.53, 0.97)) and other cause of death (HR_log2_ = 0.70 (0.48, 1.03)) and between desmosterol and other cause of death (HR_log2_ = 0.86 (0.73, 1.02)), although not statistically significant. No associations with mortality outcomes were observed for the other oxysterols.

In an additional analysis, we used elastic net regression to select sets of oxysterols most strongly associated with causes of death in addition to pre-specified covariates (Additional file 1, Table S4). For the outcome cardiovascular death, five oxysterols, namely 24S-HC, 5b6b-EC, 7-KC, lanosterol, and desmosterol, were selected. The directions of effect in the combined model were similar to the analysis of individual oxysterols, however, only lanosterol remained statistically significant (HR_log2_ = 1.90 (1.22, 2.94)). For the outcome other cause of death, lanosterol and THC were selected with inverse associations for lanosterol (lanosterol, HR_log2_ = 0.68 (0.47, 0.98); THC, HR_log2_ = 1.05 (0.91, 1.22)). Only 24S-HC was selected for BC-specific death, and none of the oxysterols was selected for all-cause death or risk of recurrence. Results were similar in the sensitivity analysis excluding oxysterols with high CVs, with lanosterol showing the strongest association with cardiovascular death (HR_log2_ = 2.05 (1.20, 3.51)).

Excluding outliers did not change the effect estimates meaningfully (< 10% difference for most oxysterols; *n* ≤ 13 outliers). For desmosterol, associations with other cancer death (HR_log2_ = 1.02 (0.81, 1.27) to HR_log2_ = 0.83 (0.59, 1.17)) and with cardiovascular death (HR_log2_ = 0.95 (0.76, 1.19) to HR_log2_ = 1.25 (0.80, 1.96)) changed slightly after exclusion of outliers (*n* = 30). Additionally adjusting associations with 7-DC for chemotherapy and associations with THC for endocrine therapy and chemotherapy changed the results minimally (< 10% difference).

In the analyses stratified by outcomes occurring with < 5 years and ≥ 5 years of diagnosis (Additional file 1, Table S5), overall results were qualitatively similar in the two strata. Some associations were stronger and only statistically significant in the < 5-year survival analysis compared with the ≥ 5-year survival analysis (e.g., 5b6b-EC and cardiovascular death, < 5 years: HR_log2_ = 1.65 (1.05, 2.58) vs. ≥ 5 years: HR_log2_ = 1.36 (0.93, 1.99); lanosterol and other cancer death, < 5 years: HR_log2 _= 0.44 (0.22, 0.90) vs. ≥ 5 years: HR_log2_ = 0.79 (0.56, 1.11); lanosterol and cardiovascular death, < 5 years: HR_log2_=3.38 (1.71, 6.69) vs. ≥ 5 years: HR_log2 _**=** 1.44 (0.90, 2.30)). Other associations were weaker in the < 5 years vs. ≥ 5-year survival analysis (e.g., 7-KC and cardiovascular death < 5 years: HR_log2_ = 1.19 (0.85, 1.66) vs. ≥ 5 years: HR_log2_ = 1.31 (1.01, 1.70)). None of the oxysterols were statistically significantly associated with all-cause death, BC-specific death, or risk of recurrence in the stratified analysis.

After adjusting for multiple comparisons using Bonferroni correction for 60 tests, none of the associations were statistically significant.

## Discussion

In this large breast cancer patient cohort study, we investigated associations with causes of death for a panel of circulating oxysterols, many explored for the first time in breast cancer patients, providing novel evidence on potential prognostic factors with respect to survival following breast cancer. Using competing risks models, we observed associations between circulating oxysterols and survival outcomes following breast cancer with higher levels of circulating oxysterols, specifically 24S-HC, 5a6a-EC, 5b6b-EC, 7-KC, THC, and lanosterol, associated with a higher risk of cardiovascular death. Higher levels of circulating lanosterol were associated with a lower risk of other cancer death, and higher levels of lanosterol and desmosterol were associated with a lower risk of other causes of death. Results were similar among participants with hormone-receptor-positive breast cancer.

### Oxysterols and breast cancer recurrence and death

In prior studies of our working group, we evaluated associations between the oxysterols 27-HC and 25-HC, identified as endogenous estrogen-receptor modulators, and breast cancer risk and survival. In a nested case-control study including 530 incident breast cancer cases and 1036 controls from the EPIC Heidelberg cohort, inverse associations between circulating 27-HC and breast cancer risk were observed [17], while differential effects were observed between circulating 27-HC and 25-HC and survival in women diagnosed with breast cancer depending on the circulating estradiol levels [18]. Due to the intriguing findings for ER modulators 27-HC and 25-HC with respect to breast cancer prognosis, we aimed to extend this line of enquiry by investigating a range of other oxysterols representing different pathways of cholesterol metabolism among women with a breast cancer diagnosis and with mechanisms beyond ER signaling. No associations were observed between the selected oxysterols and breast cancer outcomes in the present study. Results were similar after controlling for the previously investigated biomarkers 27-HC, 25-HC, and estradiol. Another study investigating associations between a panel of free oxysterols and breast cancer outcomes in 58 breast cancer patients reported an association between higher levels of free THC and poorer disease-free survival (*p* = 0.037, effect estimate not reported), while no significant associations were observed for other oxysterols including 7a-HC, 7-KC, 5a6a-EC, and 5b6b-EC [19]; we observed no associations between these oxysterols and outcomes with the exception of an association between THC and recurrence in participants with hormone-receptor negative disease**.** More experimental and human studies are needed to further investigate circulating oxysterols by treatment and other factors in breast cancer.

### Oxysterols and causes of death other than breast cancer

Non-breast cancer-related deaths are of increasing importance among women with breast cancer diagnosis, particularly with improved survival rates due to early diagnosis and improved treatment [4, 26, 27]. Several studies showed an increased risk of cardiovascular disease-related mortality among women with a breast cancer diagnosis as compared to women without breast cancer [4, 5]. In line with a recent population-based study in the United States [3], we observed death due to other cancers and due to cardiovascular diseases as the leading causes of death after breast cancer in breast cancer patients of the current study.

We observed that higher circulating levels of 24S-HC, 5a6a-EC, 5b6b-EC, 7-KC, THC, and lanosterol were associated with an increased risk of cardiovascular death. Three of these oxysterols were strongly correlated with each other (*r* ≥ 0.74: 5a6a-EC, 5b6b-EC, 7-KC) and THC was moderately correlated with these three oxysterols (0.45 ≥ *r* ≥ 0.48). 24S-HC and lanosterol were moderately correlated with each other (*r* = 0.41) but were only weakly correlated with any of the other oxysterols associated with cardiovascular death (*r* ≤│0.2│). The majority of the oxysterols associated with an increased risk of cardiovascular death are downstream metabolites of cholesterol, while among the four evaluated cholesterol precursors, only high lanosterol levels were associated with increased risk of cardiovascular death.

A number of experimental studies have evaluated the role of oxysterols and plaque formation and atherosclerosis (reviewed in [28–30]); they observed cytotoxic and inflammatory effects of oxysterols through a number of mechanisms including cell apoptosis (25-HC, 7-KC, 7b-HC), induction and promotion of inflammatory processes (7-KC, 7a-HC, 27-HC, 25-HC), upregulation of p53 (7-KC, 7b-HC), and more [28]. Several studies reported higher levels of 7b-HC and 7-KC in human atherosclerotic plaques and a potential role of oxysterols in the formation of atherosclerotic lesions [29, 31]. Other effects of oxysterols include modulation of the immune system, which may also play a role in the development of cardiovascular events [32]. The current study supports the findings of experimental studies for 7-KC, 5,6-EC, and 24S-HC, and the associations observed for these oxysterols were not impacted by adjustment for 27-HC and 25-HC.

Few human studies to date have evaluated associations between oxysterols and cardiovascular disease. These studies reported associations between oxysterols including 7b-HC, 7-KC, and 5,6-EC and cardiovascular diseases and death when comparing patients with coronary artery disease to participants with normal coronary arteries [33–36]. 24S-HC from subcutaneous and visceral adipose tissue was correlated with metabolic factors including insulin, leptin, cholesterol, and hs-CRP in women with a median BMI of 33.5 kg/m^2^ (interquartile range: 29.0, 38.6) [37]. A nested case-control study reported a higher risk of cardiovascular-related events among breast cancer patients as compared to matched controls over an average follow-up period of seven years [38]. However, to our knowledge, no study to date investigated associations with oxysterols and cardiovascular mortality in women diagnosed with breast cancer.

Whether oxysterols have a direct effect on cardiovascular health in women with breast cancer by modulation of physiological processes as described above, or whether they serve as markers for oxidative stress in women with cardiovascular diseases needs to be elucidated.

Previous studies reported that breast cancer treatment including anthracyclines [39], trastuzumab [40], and radiotherapy [41] increases the risk of cardiotoxicity and cardiovascular mortality in women with breast cancer, while the effect of endocrine therapy on cardiovascular outcomes is less conclusive [3, 42, 43]. To our knowledge, there is no evidence of an association between chemotherapy and radiotherapy and oxysterol concentrations in breast cancer patients. Endocrine therapy was reported to affect oxysterol concentrations in breast cancer patients with higher levels of 5b6b-EC and 27-HC after 28 days of aromatase inhibitor treatment, lower levels of 24S-HC, 25-HC, and 7a-HC after tamoxifen treatment [44], and higher levels of 7-KC one to two years after tumor removal and endocrine therapy use [45]. In this study, only THC was strongly associated with both breast cancer treatment (chemotherapy and endocrine therapy) as well as cardiovascular mortality. However, additionally adjusting associations for these treatments did not change the results meaningfully. Radiotherapy, on the other hand, was not associated with oxysterols in this study. Whether THC may act as a mediator between chemotherapy or endocrine therapy and cardiovascular death or through other mechanisms should be further investigated.

Higher circulating levels of lanosterol were inversely associated with risk of death due to other cancers and risk of death due to causes other than cancer and cardiovascular diseases. Furthermore, we observed that associations between lanosterol and other cancer, cardiovascular, and other cause of death were stronger within the first 5 years of survival compared to longer survival; however, the number of observed outcomes in the 5-year survival analysis was small (*n* = 15 other cancer deaths, *n* = 22 cardiovascular deaths, *n* = 15 other cause of death). Although there is evidence of within-person stability of lanosterol over a one-year period in postmenopausal women without cancer (Spearman correlation, *r* = 0.66) [46], there are limited data on longer-term within-person stability of this analyte.

To our knowledge, lanosterol has not previously been evaluated with respect to cancer progression or with respect to cardiovascular death in humans. The observed inverse associations between lanosterol and other cancer death and death due to causes other than cancer and cardiovascular diseases, and the positive association observed between lanosterol and cardiovascular death are somewhat challenging to interpret. Specific to cardiovascular diseases, circulating LDL-cholesterol is a risk factor for these outcomes [47], and since lanosterol serves as marker for endogenous cholesterol synthesis [14], this may explain the positive association with risk of cardiovascular death observed here. In *in vitro* models, lanosterol accumulation has been linked to reduced interferon (IFN) type I-mediated cytokine expression [48]. Type I interferons have multiple roles, with anti-tumor and immunoregulatory functions, and may be pro- or anti-inflammatory depending on the context (e.g., divergent roles in different auto-immune diseases) [49], supporting potentially different actions related to the underlying pathologies linked to the mortality outcomes investigated in this study. To our knowledge, potential differential effects of lanosterol itself on different tissue types have not been reported, though there is evidence for tissue-dependent effects of other intermediates on the cholesterol metabolism pathway such as 27-HC as an endogenous SERM [50]. However, it should be noted that further research on lanosterol is needed to elucidate the underlying mechanisms for the associations observed in this study.

Overall, this study provides novel evidence on underlying pathways potentially playing a role in the etiology of cardiovascular mortality in women diagnosed with breast cancer, thus contributing to a better understanding of the underlying biological mechanism and providing novel avenues for future research in this area. As this study is exploratory and the first of its kind, further studies are needed to elaborate the associations between these biomarkers and cardiovascular mortality in breast cancer survivors, as well as their clinical potential.

### Limitations

We observed relatively high CVs for some of the evaluated oxysterols (5a6a-EC, 24-DHLan, 7-KC, 7b-HC), likely resulting in exposure misclassification, though it is important to note that the concentrations in the quality control samples for these oxysterols were substantially lower than those observed in the cohort samples. While we expect this is most likely a source of random error, results of oxysterols such as 7-KC and THC should be interpreted with caution. As we only had blood collected at recruitment, we were not able to observe any time-varying effects of the oxysterols. While oxysterols were further reported to play a role in neurodegenerative diseases, including Alzheimer’s disease [32, 51], we could not evaluate other outcomes of death in this study due to the limited number of events. Given the novelty of the biomarkers evaluated in the study and the lack of existing data on their associations with cardiovascular disease, we cannot assess whether the associations observed with cardiovascular death in the current study are specific to women with a breast cancer diagnosis, or more general markers of risk of cardiovascular mortality. Further studies investigating oxysterol and cardiovascular mortality both in a breast cancer and non-breast cancer population are warranted. Finally, although we made multiple comparisons, we interpret our results cautiously and in the context of biologically driven hypotheses for the associations observed, and further acknowledge that additional studies are required to confirm the observed associations.

## Conclusion

We provide first evidence on a range of circulating oxysterols and mortality following a breast cancer diagnosis. While no associations for oxysterols with breast cancer outcomes and all-cause mortality were observed, higher levels of circulating 24S-HC, 5a6a-EC, 5b6b-EC, 7-KC, THC, and lanosterol were associated with an increased risk of cardiovascular death among women diagnosed with breast cancer. Further studies are needed to investigate the associations between oxysterols and breast cancer outcomes and other causes of death both in experimental models and in observational studies to better understand the underlying biological mechanisms and the associations with other causes of death in women with a breast cancer diagnosis.

### Supplementary Information

**Additional file 1:** **Table S1.** Analyte concentrations of circulating oxysterols and coefficients of variation (CV%): MARIE patient cohort. **Table S2.** Spearman partial correlation of oxysterols and 27-HC, 25-HC, and estradiol. **Table S3.** Oxysterols and survival and recurrence following a breast cancer diagnosis restricted to ER/PR-negative cases: MARIE patient cohort. **Table S4.** Circulating oxysterols and survival and recurrence following a breast cancer diagnosis with variable selection using elastic net regression: MARIE patient cohort. **Table S5.** Oxysterols and survival and recurrence following a breast cancer diagnosis stratified by 5 years of follow-up.

## Data Availability

The datasets used and/or analyzed during the current study are available from the corresponding author on reasonable request.

## Abbreviations

22R-HC: 22R-hydroxycholesterol
24,25-EC: 24,25-Epoxycholesterol
24-DHLan: 24,25-Dihydrolanosterol
24S-HC: 24S-Hydroxycholesterol
25-HC: 25-Hydroxycholesterol
27-HC: 27-Hydroxycholesterol
5a6a-EC: 5α,6α-Epoxycholesterol
5b6b: 5β,6β-Epoxycholesterol
7a-HC: 7α-Hydroxycholesterol
7b-HC: 7β-Hydroxycholesterol
7-DC: 7-Dehydrocholesterol
7-KC: 7-Ketocholesterol
AI: Aromatase inhibitor
BC: Breast cancer
BMI: Body mass index
CCI: Charlson Comorbidity Index
CI: Confidence interval
CV: Coefficient of variation
desmos: Desmosterol
ER: Estrogen receptor
FU: Follow-up
HER2: Human epidermal growth factor 2
HR: Hazard ratio
lan: Lanosterol
LOD: Limit of detection
nM: Nanomolar
PR: Progesterone receptor
SERM: Selective estrogen receptor modulator
THC: 5α,6β-Dihydroxycholestanol
