# Supplementary material for: Circulating oxysterols and prognosis among women with a breast cancer diagnosis: results from the MARIE patient cohort
Source: BMC Med. 2023 Nov 14;21:438. doi: 10.1186/s12916-023-03152-7 (PMC10648629; doi:10.1186/s12916-023-03152-7)
Supplement: Supplementary file 1 — Additional file 1: Table S1. Analyte concentrations of circulating oxysterols and coefficients of variation (CV%): MARIE patient cohort. Table S2. Spearman partial correlation of oxysterols and 27-HC, 25-HC, and estradiol. Table S3. Oxysterols and survival and recurrence following a breast cancer diagnosis restricted to ER/PR-negative cases: MARIE patient cohort. Table S4. Circulating oxysterols and survival and recurrence following a breast cancer diagnosis with variable selection using elastic net regression: MARIE patient cohort. Table S5. Oxysterols and survival and recurrence following a breast cancer diagnosis stratified by 5 years of follow-up. [file 12916_2023_3152_MOESM1_ESM.docx]

# Supplementary information

### Table S1: Analyte concentrations of circulating oxysterols and coefficients of variation (CV%): MARIE patient cohort

| Analyte (nM) | N (imputed) | <LOD | >calibration | Median (range) study population in nM | Inter-assay CV % | Intra-assay CV % |
| --- | --- | --- | --- | --- | --- | --- |
| *Exposure analytes* | |  |  |  |  |  |
| 24S-HC | 2280 | 7 | 2 | 94.3 (17.2-1973) | 20.2 % | 7.9 % |
| 7a-HC | 2271 | - | 11 | 272.2 (25.6-5458) | 17.3% | 9.5 % |
| 7b-HC* | 2272 | 14 | 10 | 201.2 (1.3-6061) | 74.9% | < LOD |
| 7-KC | 2282 | - | - | 184.9 (10.1-106000) | 42.4% | 9.1 % |
| 5a6a-EC | 2281 | - | 1 | 26.3 (1.8-4559) | 31.8 % | 18.4 % |
| 5b6b-EC | 2278 | - | 4 | 101.3 (5.9-10228) | 20.0% | 7.6 % |
| THC | 2282 | 492 | - | 7.1 (0.3-758) | 41.3% | < LOD |
| 7-DC | 2221 | 150 | 61 | 541.7 (9.6-10252) | 18.3% | 9.8 % |
| 24-DHLan** | 1027 | 1255 | - | 40.1 (5.6-413) | 33.7 % | < LOD |
| Lan | 2282 | - | - | 545.2 (57.8-3256) | 20.7% | 14.2 % |
| Desmos | 2281 | 11 | 1 | 1875.0 (51.9-10643) | 20.2% | 6.5 % |
| 22R-HC** | 281 | 2001 | - | 6.4 (1.4-142) | < LOD | < LOD |
| 24, 25-EC** | 90 | 2192 | - | 11.5 (5.8-105) | < LOD | < LOD |
| *Covariate analytes* | |  |  |  |  |  |
| 27-HC | 2282 | - | - | 210.0 (85.6-600) | 15.8% | 7.5 % |
| 25-HC | 2282 | 89 | - | 20.4 (2.5-5719) | 29.2% | 16.0 % |
| Estradiol*** | 2282 | 12 | - | 0.08 (0.0-4.3) | 16.2% | 13.8% |

N= total number of biomarker values including imputed values and excluding values >calibration range; unit: nM=nanomolar
<LOD=values below the limit of detection (LOD), imputed with the midpoint between 0 and the lowest detectable value.
>calibration=values exceeding calibration range, excluded from main analysis
outliers=outliers after imputation and exclusion of values > calibration range.

*Excluded in main analysis due to high CV% but highly correlated “partner-analyte” (7a-HC) included
**No imputation due to high proportion (>50%) of values below LOD
***Estradiol: 0.08 (0.0-4.3) nM corresponding to 22.1 (0.0-1175) ng/ml

Abbreviations: 24S-HC=24S-hydroxycholesterol; 5a6a-EC=5α,6α-epoxycholesterol; 5b6b-EC=5β,6β-epoxycholesterol; 7-KC=7-ketocholesterol; 7a-HC=7α-hydroxycholesterol; 7b-HC=7β-hydroxycholesterol; Lan=lanosterol; 24-DHLan=24,25-dihydrolanosterol; 7-DC=7-dehydrocholesterol; Desmos=desmosterol; THC=5α,6β-dihydroxycholestanol; 24,25-EC=24,25-epoxycholesterol; 22R-HC=22R- hydroxycholesterol; 27-HC=27-hydroxycholesterol; 25-HC=25-hydroxycholesterol.

### Table S2: Spearman partial correlation of oxysterols and 27-HC, 25-HC, and estradiol

|  | **24S-HC** | **7a-HC** | **7b-HC** | **7-KC** | **5a6a-EC** | **5b6b-EC** | **THC** | **7-DC** | **Lan** | **24-DHLan** | **Desmos** | **22R-HC** | **24,25-EC** |
| --- | --- | --- | --- | --- | --- | --- | --- | --- | --- | --- | --- | --- | --- |
| *n* | *2269* | *2269* | *2269* | *2269* | *2269* | *2269* | *2269* | *2209* | *2209* | *1019* | *2269* | *263* | *86* |
| **27-HC** | 0.51 | 0.16 | 0.13 | 0.14 | 0.26 | 0.26 | 0.20 | 0.23 | 0.42 | 0.32 | 0.28 | 0.22 | 0.01 |
| **25-HC** | 0.32 | 0.44 | 0.47 | 0.50 | 0.45 | 0.45 | 0.39 | 0.10 | 0.15 | 0.20 | 0.04 | 0.29 | -0.08 |
| **Estradiol** | -0.01 | 0.01 | -0.002 | 0.04 | 0.01 | 0.03 | 0.01 | -0.05 | -0.11 | -0.01 | -0.10 | 0.07 | -0.29 |

Spearman partial correlation coefficients, adjusted for age and center; using imputed, log2-transformed values. Abbreviations: 27-HC=27- hydroxycholesterol ; 25-HC=25- hydroxycholesterol; 24S-HC=24S-hydroxycholesterol; 7a-HC=7α-hydroxycholesterol; 7b-HC=7β-hydroxycholesterol; ; 7-KC=7-ketocholesterol; 5a6a-EC=5α,6α-epoxycholesterol; 5b6b-EC=5β,6β-epoxycholesterol; THC=5α,6β-dihydroxycholestanol; 7-DC=7-dehydrocholesterol; Lan=lanosterol; 24-DHLan=24,25-dihydrolanosterol; Desmos=desmosterol; 22R-HC=22R- hydroxycholesterol; 24,25-EC=24,25-epoxycholesterol.

### Table S3: Oxysterols and survival and recurrence following a breast cancer diagnosis restricted to ER/PR-negative cases: MARIE patient cohort

|  |  | All-cause death | | BC-specific death | | | | |  | | | Recurrence |
| --- | --- | --- | --- | --- | --- | --- | --- | --- | --- | --- | --- | --- |
|  | **n/events** | **HR (95% CI)** | | **n/events** | | **HR (95% CI)** | | | **n/events** | | **HR (95% CI)** | |
| *Cholesterol metabolites* | | |  | |  | |  |  | |  |  |  |
| 24S-HC | *320/88* | 1.06 (0.65,1.74) | | *320/58* | | 0.92 (0.53,1.58) | | | *320/85* | | 1.40 (0.84,2.36) | |
| 7a-HC | *319/87* | 0.72 (0.55,0.94) | | *319/57* | | 0.75 (0.54,1.04) | | | *319/84* | | 0.95 (0.72,1.25) | |
| 7-KC | *320/88* | 0.90 (0.75,1.07) | | *320/58* | | 0.90 (0.72,1.13) | | | *320/85* | | 1.02 (0.85,1.23) | |
| 5a6a-EC | *320/88* | 1.00 (0.77,1.30) | | *320/58* | | 1.01 (0.73,1.40) | | | *320/85* | | 1.16 (0.89,1.50) | |
| 5b6b-EC | *320/88* | 0.93 (0.73,1.18) | | *320/58* | | 0.97 (0.71,1.32) | | | *320/85* | | 1.10 (0.86,1.42) | |
| THC | *320/88* | 1.05 (0.92,1.20) | | *320/58* | | 1.32 (0.95,1.83) | | | *320/85* | | 1.38 (1.05,1.81) | |
| *Cholesterol precursors* | |  | |  | |  | | |  | |  | |
| 7-DC | *313/87* | 1.01 (0.88,1.15) | | *313/58* | | 1.07 (0.94,1.22) | | | *313/85* | | 1.01 (0.92,1.11) | |
| Lan | *320/88* | 0.97 (0.72,1.31) | | *320/58* | | 0.93 (0.65,1.34) | | | *320/85* | | 0.92 (0.67,1.25) | |
| 24-DHLan | *161/45* | 0.87 (0.59,1.30) | | *161/29* | | 0.94 (0.57,1.54) | | | *161/43* | | 0.84 (0.56,1.26) | |
| Desmos | *320/88* | 1.16 (0.86,1.58) | | *320/58* | | 1.11 (0.82,1.50) | | | *320/85* | | 1.31 (0.93,1.84) | |

Hazard ratios (HR), 95% confidence intervals (95% CI), and p-het from competing risks models. All models are adjusted for age at diagnosis, BMI, tumor size, nodal status, histological grading, smoking status (never, former, current), alcohol consumption, Charlson Comorbidity Index (CCI), and stratified by study region. Oxysterol values are log2-transformed.
Abbreviations: 24S-HC=24S-hydroxycholesterol; 22R-HC=22R-hydroxycholesterol; 5a6a-EC=5α,6α-epoxycholesterol; 5b6b-EC=5β,6β-epoxycholesterol; 7-KC=7-ketocholesterol; 7a-HC=7α-hydroxycholesterol; Lan=lanosterol; 24-DHLan=24,25-dihydrolanosterol; 7-DC=7-dehydrocholesterol; Desmos=desmosterol; THC=5α,6β-dihydroxycholestanol.

### **Table S4: Circulating oxysterols and** survival and recurrence following a breast cancer diagnosis **with** variable selection using elastic net regression: MARIE patient cohort

|  | **N total /n events** | **Per 1 unit increase HR (95% CI)** |
| --- | --- | --- |
| **All oxysterols** |  |  |
| **All-cause death** |  |  |
| *None selected* |  |  |
| **BC-specific death** |  |  |
| 24S-HC | 2263/236 | 1.22 (0.93, 1.61) |
| **Other cancer death** |  |  |
| Lan | 2265/87 | 0.70 (0.52,0.95) |
| **Cardiovascular death** |  |  |
| 24S-HC | 2263/60 | 1.13 (0.61, 2.09) |
| 5b6b-EC | 2261/60 | 1.13 (0.62, 2.06) |
| 7-KC | 2265/60 | 1.13 (0.75, 1.70) |
| Lan | 2265/60 | 1.90 (1.22, 2.94) |
| Desmos | 2264/60 | 0.78 (0.59, 1.04) |
| **Other cause of death** |  |  |
| Lan | 2265/51 | 0.68 (0.47, 0.98) |
| THC | 2265/51 | 1.05 (0.91, 1.22) |
| **Recurrence** |  |  |
| *None selected* |  |  |
| **Oxysterols with low CV** |  |  |
| **All-cause death** |  |  |
| *None selected* |  |  |
| **BC-specific death** |  |  |
| 24S-HC | 2263/236 | 1.22 (0.93, 1.61) |
| **Other cancer death** |  |  |
| *None selected* |  |  |
| **Cardiovascular death** |  |  |
| 24S-HC | 2263/60 | 1.17 (0.61, 2.24) |
| 5b6b-EC | 2264/60 | 1.48 (0.94, 2.35) |
| 7a-HC | 2254/60 | 0.82 (0.52, 1.29) |
| 7-DC | 2205/56 | 0.92 (0.74, 1.13) |
| Lan | 2265/60 | 2.05 (1.20, 3.51) |
| Desmos | 2264/60 | 0.84 (0.52, 1.38) |
| **Other cause of death** |  |  |
| *None selected* |  |  |
| **Recurrence** |  |  |
| *None selected* |  |  |

Automatic variable selection using regularized cox proportional hazard models with elastic net penalty. Hazard ratios (HR) and 95% confidence intervals (95% CI) from Cox proportional hazard models. No 24-DHLan due to missings

All models are adjusted for age at diagnosis, BMI, tumor size, nodal status, histological grading, smoking status, alcohol consumption, Charlson Comorbidity Index (CCI, 0, 1, 2+), and stratified by study region and ER/PR-status. p_trend_ for the median of quartiles. Quartiles were calculated using the center-specific distribution. A 1-unit increase in the log_2_ transformed oxysterol concentration corresponds to a doubling. Abbreviations: 27-HC=27-hydroxycholesterol; 25-HC=25-hydroxycholesterol; 24S-HC=24S-hydroxycholesterol; 22R-HC=22R-hydroxycholesterol; 5a6a-EC=5α,6α-epoxycholesterol; 5b6b-EC=5β,6β-epoxycholesterol; 7-KC=7-ketocholesterol; 7a-HC=7α-hydroxycholesterol; 7b-HC=7β-hydroxycholesterol; Lan=lanosterol; 24-DHLan=24,25-dihydrolanosterol; 7-DC=7-dehydrocholesterol; Desmos=desmosterol; THC=5α,6β-dihydroxycholestanol.

### Table S5: Oxysterols and survival and recurrence following a breast cancer diagnosis stratified by 5 years of follow-up

|  |  |  | Follow up time < 5 years | | | |  |
| --- | --- | --- | --- | --- | --- | --- | --- |
|  | n total | **All-cause death**  **HR (95% CI)*,  n=139** | **BC-specific death**  **HR (95% CI),  n=87** | **Other cancer death**  **HR (95% CI),**  **n=15** | **Cardiovascular death,**  **HR (95% CI),  n=22** | **Other death,**  **HR (95% CI),  n=15** | **Recurrences,**  **HR (95% CI),  n=90** |
| *Cholesterol metabolites* | | |  |  |  |  |  |
| 24S-HC | *2263* | 1.13 (0.78,1.63) | 1.08 (0.69,1.68) | 0.92 (0.32,2.68) | 1.80 (0.79,4.10) | 0.84 (0.35,2.02) | 1.22 (0.78,1.91) |
| 7a-HC | *2254* | 0.94 (0.76,1.16) | 0.93 (0.72,1.22) | 0.88 (0.46,1.68) | 1.00 (0.58,1.72) | 1.18 (0.62,2.25) | 0.95 (0.73,1.23) |
| 7-KC | *2265* | 0.96 (0.83,1.11) | 0.88 (0.73,1.06) | 1.13 (0.76,1.70) | 1.19 (0.85,1.66) | 1.09 (0.71,1.67) | 0.90 (0.75,1.08) |
| 5a6a-EC | *2264* | 0.96 (0.78,1.19) | 0.90 (0.68,1.18) | 1.12 (0.60,2.09) | 1.31 (0.85,2.02) | 0.80 (0.40,1.58) | 0.93 (0.71,1.21) |
| 5b6b-EC | *2261* | 0.97 (0.79,1.18) | 0.85 (0.65,1.10) | 1.10 (0.58,2.09) | **1.65 (1.05,2.58)** | 0.85 (0.47,1.55) | 0.88 (0.68,1.14) |
| THC | *2265* | 1.11 (1.00,1.22) | 1.08 (0.84,1.40) | 1.28 (0.76,2.18) | 1.20 (0.78,1.86) | 1.39 (0.83,2.33) | 1.15 (0.90,1.46) |
| *Cholesterol precursors* | | |  |  |  |  |  |
| 7-DC | *2205* | 1.00 (0.90,1.12) | 1.00 (0.91,1.10) | 0.88 (0.74,1.05) | 1.09 (0.84,1.42) | 0.86 (0.73,1.01) | 1.01 (0.92,1.11) |
| Lan | *2265* | 0.90 (0.71,1.13) | 0.87 (0.65,1.17) | **0.44 (0.22,0.90)** | **3.38 (1.71,6.69)** | **0.38 (0.20,0.74)** | 0.89 (0.66,1.19) |
| 24-DHLan | *1023* | 1.07 (0.77,1.47) | 0.87 (0.58,1.31) | 1.16 (0.48,2.79) | 2.29 (0.98,5.35) | 0.71 (0.26,1.94) | 0.85 (0.57,1.28) |
| Desmos | *2264* | 0.94 (0.78,1.14) | 1.02 (0.83,1.25) | 0.84 (0.62,1.14) | 1.23 (0.66,2.29) | 0.82 (0.62,1.08) | 1.03 (0.84,1.26) |
| Follow up time ≥ 5 years | | | | | | | |
|  | n total | **All-cause death**  **HR (95% CI)*,  n=295** | **BC-specific death**  **HR (95% CI),  n=150** | **Other cancer death**  **HR (95% CI),**  **n=73** | **Cardiovascular death,**  **HR (95% CI),  n=38** | **Other death,**  **HR (95% CI),  n=43** | **Recurrences,**  **HR (95% CI),  n=286** |
| *Cholesterol metabolites* | | |  |  |  |  |  |
| 24S-HC | *2263* | 1.11 (0.86,1.42) | 1.34 (0.96,1.87) | 0.72 (0.44,1.18) | 1.63 (0.83,3.20) | 0.89 (0.47,1.70) | 1.16 (0.91,1.49) |
| 7a-HC | *2254* | 0.99 (0.86,1.15) | 0.97 (0.80,1.19) | 0.86 (0.65,1.16) | 1.33 (0.89,1.98) | 1.17 (0.81,1.70) | 0.88 (0.76,1.03) |
| 7-KC | *2265* | 1.05 (0.95,1.15 | 1.03 (0.90,1.17) | 0.94 (0.77,1.14) | **1.31 (1.01,1.70)** | 1.08 (0.85,1.38) | 0.96 (0.87,1.06) |
| 5a6a-EC | *2264* | 1.07 (0.93,1.22) | 1.02 (0.85,1.24) | 0.99 (0.74,1.32) | 1.35 (0.93,1.96) | 1.13 (0.79,1.61) | 0.97 (0.84,1.12) |
| 5b6b-EC | *2261* | 1.11 (0.97,1.27) | 1.05 (0.87,1.28) | 1.00 (0.75,1.33) | 1.36 (0.93,1.99) | 1.23 (0.87,1.75) | 0.99 (0.86,1.14) |
| THC | *2265* | 1.01 (0.95,1.07) | 0.98 (0.90,1.07) | 1.06 (0.93,1.21) | 1.16 (0.94,1.43) | 0.98 (0.84,1.14) | 0.98 (0.92,1.04) |
| *Cholesterol precursors* | | |  |  |  |  |  |
| 7-DC | *2205* | 0.98 (0.91,1.06) | 0.97 (0.88,1.09) | 0.97 (0.84,1.13) | 1.02 (0.83,1.26) | 0.93 (0.77,1.13) | 0.95 (0.88,1.02) |
| Lan | *2265* | 1.03 (0.87,1.22) | 1.10 (0.86,1.40) | 0.79 (0.56,1.11) | 1.44 (0.90,2.30) | 0.93 (0.59,1.45) | 1.06 (0.89,1.27) |
| 24-DHLan | *1023* | 0.89 (0.72,1.10) | 0.91 (0.68,1.24) | 0.87 (0.56,1.36) | 0.70 (0.39,1.26) | 0.90 (0.53,1.53) | 1.00 (0.79,1.25) |
| Desmos | *2264* | 1.03 (0.88,1.20) | 1.09 (0.86,1.37) | 1.09 (0.78,1.52) | 0.90 (0.62,1.30) | 0.84 (0.60,1.17) | 1.15 (0.97,1.37) |

*Hazard ratios (HR) and 95% confidence intervals (95% CI) for all-cause mortality from Cox proportional hazard models, with <5 year and ≥ 5 year follow-up time. All other HR and 95% CI from competing risks models, with 5-year follow-up time. All models are adjusted for age at diagnosis, BMI, tumor size, nodal status, histological grading, smoking status (never, former, current), alcohol consumption, Charlson Comorbidity Index (CCI), and stratified by study region and ER/PR-status. Oxysterol values are log_2_-transformed.
Abbreviations: 24S-HC=24S-hydroxycholesterol; 22R-HC=22R-hydroxycholesterol; 5a6a-EC=5α,6α-epoxycholesterol; 5b6b-EC=5β,6β-epoxycholesterol; 7-KC=7-ketocholesterol; 7a-HC=7α-hydroxycholesterol; Lan=lanosterol; 24-DHLan=24,25-dihydrolanosterol; 7-DC=7-dehydrocholesterol; Desmos=desmosterol; THC=5α,6β-dihydroxycholestanol.
